# Supplementary material for: The Males Absent on the First (MOF) Mediated Acetylation Alters the Protein Stability and Transcriptional Activity of YY1 in HCT116 Cells
Source: Int J Mol Sci. 2023 May 13;24(10):8719. doi: 10.3390/ijms24108719 (PMC10217912; doi:10.3390/ijms24108719)
Supplement: Supplementary file 1 [file ijms-24-08719-s001.zip › ijms-2388158-supplementary.pdf]

Figure S1

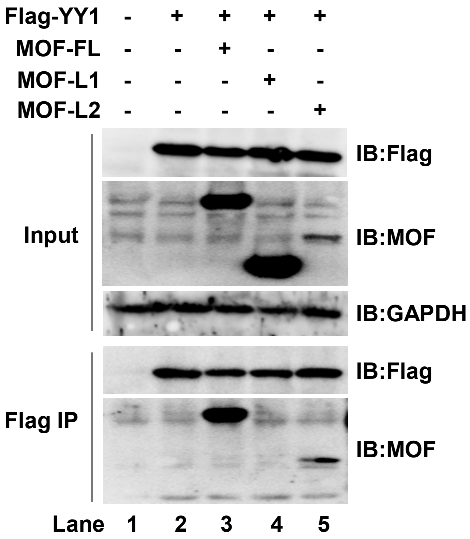

Figure S1. YY1 binds to the C-terminal of MOF. Co-transfection of YY1 with MOF full-length, N-terminal (1-157 aa) or C-terminal (158-459 aa) region of MOF into HCT116 cells. 48 hours later, cells were collected and whole cell lysate were prepared. Bound truncated MOF proteins were detected with western blot following Flag IP.

Figure S2

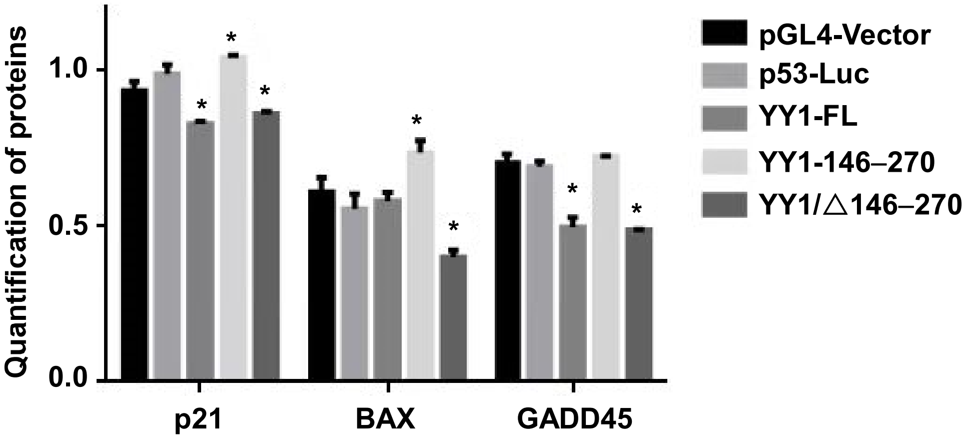

Figure S2. Quantified protein levels of p21、Bcl2、GADD45 (normalized to GAPDH).
* p <0.05, compared to the corresponding pGL4-Vector group.

Figure S3

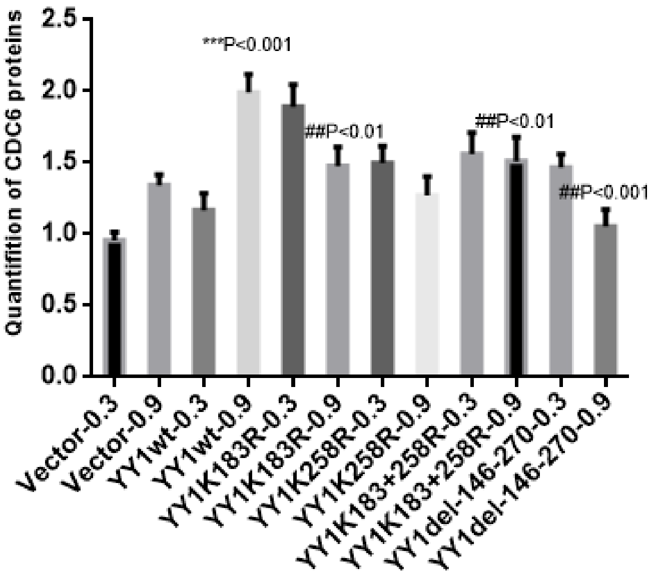

Figure S3. Quantitative analysis of CDC6 proteins (normalized to GAPDH). ***$p < 0.001$, compared to empty vector group, ##$p < 0.01$ compared to YY1wt group.
